# Gender-based violence screening methods preferred by women visiting a public hospital in Pune, India

**DOI:** 10.1186/s12905-018-0515-2

**Published:** 2018-01-15

**Authors:** Nishi Suryavanshi, Shilpa Naik, Smita Waghmare, Nikhil Gupte, Sameer Khan, Vidya Mave, Andrea Deluca, Amita Gupta, Jonathan Golub, Robert C. Bollinger, Anita Shankar

**Affiliations:** 1 BJ Government Medical College - Johns Hopikns University Clinical Trial Unit, Jai Prakash Narayan Road, Pune, 411001 India; 2 BJ Government Medical College, Department of Obstetrics and Gynaecology, Jai Prakash Narayan Road, Pune, India; 3 Johns Hopkins University, School of Medicine, Baltimore, USA; 4 Johns Hopkins University, Bloomberg School of Public Health, Baltimore, USA

**Keywords:** Women and violence, Gender based violence, Screening, Face-to-face interview

## Abstract

**Background:**

Gender-based violence (GBV) is a major global public health concern and is a risk factor for adverse health outcomes. Early identification of GBV is crucial for improved health outcomes. Interactions with health care providers may provide a unique opportunity for routine GBV screening, if a safe, confidential environment can be established.

**Methods:**

Between November 2014 and February 2015, a cross-sectional, observational study was conducted where women were interviewed about their opinions concerning GBV screening in a tertiary health care setting in Pune, India. Trained counsellors interviewed 300 women at different out-patient and in-patient departments using a semi-structured questionnaire.

**Results:**

Twenty-three percent of these women reported experiencing GBV in their life. However, 90% of women said they had never been asked about GBV in a health care setting. Seventy-two percent expressed willingness to be asked about GBV by their health care providers, with the preferred provider being nurses or counsellors. More than half (53%) women reported face-to-face interview as the most preferred method for screening. There were no major differences in these preferences by GBV history status.

**Conclusions:**

Our study provides evidence for preferred GBV screening methods and optimal provider engagement as perceived by women attending a public hospital.

**Electronic supplementary material:**

The online version of this article (10.1186/s12905-018-0515-2) contains supplementary material, which is available to authorized users.

## Background

Globally, one in three women (35.6%) report having experienced physical and/or sexual partner violence, or sexual violence by a non-partner [1]. In India, 37% of women report lifetime physical, sexual, or psychological abuse also referred to as gender based violence (GBV) [2]. In Maharashtra, the estimate of GBV prevalence is 31% [3], and in Pune district, with a population of 9 million, GBV has been reported by 10%, [4] of women interviewed in a health care setting and up to 62% among women in urban slums [5].

There are multiple challenges to identifying at-risk women and uncertainty about how and where screening should take place [6]. GBV screening at health care facilities is already a well-established part of routine care in developed countries, where health facilities can provide a safe, confidential environment, leading to improved communication and early referral service [7].

### Objective

India has yet to adopt routine GBV screening of women in health care facilities and there are limited data on Indian women’s perceptions and acceptability of GBV screening. This study was undertaken with an objective to examine preferred GBV screening processes among women presenting to a public, urban Indian tertiary health care center.

## Methods

### Study design, settings and participants

A cross-sectional, observational study was conducted among women ≥ 18 years of age presenting to the antenatal clinic, medical termination of pregnancy outpatient department (OPD), gynaecology OPD, anti-retroviral treatment clinic, postpartum wards and postnatal clinic at BJ Government Medical College/Sassoon Hospital in Pune, between November 2014 and February 2015. Two trained counsellors conducted weekly visits to each clinic and approached an average of 3–4 consecutive women (age ≥ 18 years) per day.

Women who consented to be interviewed were enrolled in the study and a semi-structured questionnaire (Additional file 1) was administered by counsellors. We obtained a representative sample of women attending hospital wards that the counsellors were responsible to support. This was done as follows: we pre-assigned days in the week in which one counsellor was to complete 3–4 interviews in the clinic that they were serving. Once assigned, and after their daily commitments were completed, the counsellors identified one prospective woman to request and interview. If she agreed, informed consent was obtained and the interview was completed. After this point, an additional 2–3 interviews were conducted consecutively in the ward.

### Data variables and definition

Data were collected on demographics, knowledge of GBV support organisations, previous experience and acceptability of GBV screening in the health care setting, preferred method and preferred health care professional for GBV screening, perceptions of women’s willingness to honestly respond to GBV questions posed by health care providers, and personal experience of GBV.

The operational definition of gender based violence used for this study is self-reported experience of one or more acts of physical, verbal and/or sexual abuse by intimate partner at any time after her marriage.

We accept that questionnaire-based interviewing about the reporting of such behaviours is likely to result in under-reporting of these topics.

The analyses below should be interpreted in the light of this possible under-reporting.

### Statistical analysis

We used descriptive statistics to describe baseline characteristics of participants. Data were analysed using STATA version 13.1. Bivariate analysis was done to determine association between experiences of GBV and preferred methods of GBV screening, educational status, family type, acceptability of GBV screening and HIV status.

The study was reviewed and approved by the B.J. Medical College Ethics Committee and the Johns Hopkins Medicine Institutional Review Board.

## Results

Of 302 women approached by the study team, 300 with a median age of 25 years (IQR 22–30) consented for interviews. Most participating women were married, belonged to nuclear families, were literate and unemployed (Table 1).

**Table 1:** Descriptive characteristics of women stratified by experience of gender based violence in a public health care setting

*N* = 300	Overall	GBV experience = Yes N (%) 70 (23.3)	GBV experience = No N (%) 230 (69.7)	*P*-Value
Characteristics				
Age (Yrs), Median (IQR)	25 (22–30)	24 (25–28)	25 (22–30)	0.17
Marital Status				
Married	267 (89.0)	60 (86)	207(90.0)	0.35
Separated/Divorced	032 (11.0)	10 (14.3)	022(10.0)	
Family type				
Nuclear	179 (59.5)	41 (58.5)	138(60.0)	0.88
Joint	119 (39.5)	29 (41.3)	090(39.0)	
Employment status				
Employed	092 (31.0)	11 (15.7)	058 (25.0)	0.12
Not employed	207(69.0)	59(84.2)	166 (72.1)	
Educational Status				
Illiterate	058 (19.4)	11 (15.7)	047(20.4)	0.28
Primary	062 (20.7)	19 (27.0)	044(19.1)	
= > Secondary	179 (60.1)	40 (56.5)	137(59.4)	
Awareness about GBV support Organization				
Yes	067 (23.0)	21(30.5)	046(20.0)	0.05
No	226 (76.0)	48(69.5)	179 (77.8)	
GBV screening done in health care setting.				
Yes	030 (10.1)	09 (12.8)	26(11.3)	0.65
No	269 (89.9)	61(87.1)	204(88.6)	
Do women want to be asked about GBV in Hospital				
Yes	212 (71.9)	54 (77.14)	159(69.1)	0.20
No	083 (28.1)	15(21.4)	068(30.0)	
Preferred Health Provider to inquire about violence				
Doctor	009 (3.0)	03 (4.35)	006(2.6)	0.45
Counselor/Nurse	228 (76.0)	57(81.4)	171 (74.3)	
Others	055(18.3)	10 (14.2)	045(19.5)	
Preferred technique for GBV screening				
Face to face interview	247 (83.0)	53 (75.7)	194 (84.3)	0.15
Completing a survey	043 (14.3)	12 (17.1)	031(13.4)	
Computerized/mobile tool	034 (11.0)	12 (17.1)	022(09.5)	
HIV Status*				
Positive	102 (64.0)	28 (70.0)	74 (62.1)	0.45
Negative	57 (36.0)	12 (30.0)	45 (38.0)	

*HIV status: Unknown: 139 Women did not know their HIV status when interviewed for GBV

Forty-six (23%) of these women reported that they were currently or had previously experienced GBV. However, most (*n* = 269; 90%) of the women had never been previously asked about GBV in a health care setting. Notably, 61 (87%) of the 70 women reporting GBV history had never previously been asked about their risk. Two-hundred and twelve (72%) of the women expressed willingness to be asked about GBV by their health care providers and in the health care settings. The women overwhelming (85%) expressed a preference for that screening to be provided by a nurse or counsellor and that GBV screening be a face-to-face private interview (83%).

## Discussion

### Key results

The results of this study are in concordance with the findings of previous studies that gender based violence screening is acceptable by women in health care setting (15–18). In our study we found most preferred method reported is face- to-face interview and most preferred provider for GBV screening was counselor. This illustrate need for screening women in public health facilities by counselors.

### Interpretation

In our sample, we found that current or past experience with GBV was reported by about 1 in 4 Indian women attending this public hospital setting. This is lower than national averages that range between 37% [2] and 56% [8]. The reasons for this are not clear, however, may reflect the wide range of methodologies used or regional variation due to socio-cultural context [6]. These high rates of GBV require more concerted efforts to identify, screen and facilitate care for affected women, as prior studies have demonstrated significant adverse impact of GBV on health outcomes [9]. GBV in India has been shown to be associated with sexually transmitted infections, adverse reproductive health outcomes and mental health [10, 11] making it an important public health concern. Importantly, we found that among Indian women who experienced GBV, only 10% reported that they were asked about GBV when they presented to health care setting. This highlights a critical unmet need for GBV screening, care and referral for women experiencing GBV.

Routine screening for GBV is recommended as part of the standard of care within health care settings in resource rich countries [8, 12, 13] highlighting the importance of a public health approach in GBV [14]. Despite this recommendation, the implementation of GBV screening in resource-limited settings has been suboptimal. This has been very well demonstrated by our study where a majority of individuals who reported a history of GBV reported never having been asked about GBV in health care settings.

A qualitative study in US [15] suggested that women were supportive of being asked about their experience of intimate partner violence, during volunteering counselling and testing (VCT) sessions. Furthermore, a feasibility study in Kenya [16], Nigeria [17] and in India [18] demonstrated that routine GBV screening is feasible and acceptable by women presenting in public health systems. Consistent with these findings, our study demonstrated that over two-thirds of women were willing to be screened for GBV in health care settings.

Further, our results demonstrated the important role for counsellors and secondly nurses in settings like India, as in US counterparts, where over two-thirds of women prefer GBV screening to be done by nurses and doctors [19]. Nurses are universally accepted as preferred GBV providers. Very few women (3%) in our study want their treating doctor to be the person to screen for GBV. Although doctors have opportunities conduct GBV screening, training about GBV in medical colleges and hospitals is very limited or absent [20]. Since previous reports in India suggest that reporting of GBV may be lower when screened by the doctors [4], physician screening may not be optimal for Indian women and this is supported by our study.

Finally, two-thirds of women in our study chose face-to-face interview as the most preferred method for GBV screening. While none of the previous studies inquired on women’s preference for face-to-face versus e-screening options, the clear preference for face-to-face screening in our study are likely due to unfamiliarity with surveys or computerised tools, cultural norms related to stigmatization of GBV and associated shame for the victim or may be personal interaction with same gender is important for such a sensitive topic.

### Limitations

A potential limitation of our study includes bias in sample selection as only those women were approached who could give some time for the interview on days where counsellors attended the clinic. It is possible that women may underreport self-reported GBV but generally GBV prevalence is assessed using standardized questionnaire and are subject to perception bias. Moreover, due to limited time per interview, we focused on broad questions related to GBV history and experience, whereas more details may have been elucidated through a larger battery of questions. However, our study identified some key GBV screening strategies that can be employed in public health care settings.

### Generalisability

Previous studies in resource rich setting [15] and resource limited settings [16, 17] demonstrated that routine GBV screening in health settings is feasible and acceptable among women. Our study findings are in concordance with these findings. However, our study reported face to face interviews by counsellors as most preferred method and most preferred provider for GBV screening. As we did not find any study in resource limited settings that could identify preferred method and provider there is need for further studies to confirm these findings.

## Conclusion

Screening for GBV is acceptable to women and our study provides evidence for preferred provider and method for GBV screening, optimizing the opportunity to screen women within the health care system in order to identify and address this critical public health issue.

## Additional file

Additional file 1: Gender Based Violence Questionnaire. (PDF 471 kb)

## Abbreviations

GBV: Gender Based Violence
HIV: Human Immunodeficiency Virus
OPD: Out Patient Department

## References

[1] WHO DoRHaR, London School of Hygiene and Tropical Medicine, South African Medical Research Council (2013). Global and regional estimates of violence against women prevalence and health effects of intimate partner violence and non-partner sexual violence: WHO reference number: 978 92 4 156462 5.

[2] UN-Women (2011). Facts and figures on violence against women.

[3] NFHS-4. National Family Health Survey (NFHS-4). In: Macro IIfPSa, international, eds. Mumbai, 2014–2015. 2015.

[4] Salvi PPG, Bhosale R, Chandanwale A (2014). Magnitude and risk factors for physical domestic violence during pregnancy. Int J Sci Stud.

[5] Ruikar MM, Pratinidhi AK (2008). Physical wife abuse in an urban slum of Pune, Maharastra. Indian J Public Health.

[6] Coast ELT, Malviya A (2012). Gender-based violence and reproductive health in five Indian states.

[7] Warshaw C, Alpert E (1999). Integrating routine inquiry about domestic violence into daily practice. Ann Intern Med.

[8] Babu BV, Kar SK. Domestic violence against women in eastern India: a population-based study on prevalence and related issues. BMC Public Health. 2009;9:129. 10.1186/1471-2458-9-129. [published Online First: 2009/05/12] 10.1186/1471-2458-9-129 PMC2685379 19426515

[9] Koenig LJ, Whitaker DJ, Royce RA, et al. Physical and sexual violence during pregnancy and after delivery: a prospective multistate study of women with or at risk for HIV infection. Am J Public Health. 2006;96(6):1052–9. 10.2105/ajph.2005.067744. [published Online First: 2006/05/04] 10.2105/AJPH.2005.067744 PMC1470613 16670222

[10] Deosthali P, Maghnani P, Malik S (2005). Establishing Dilaasa: documenting the challenges.

[11] Chowdhary N, Patel V (2008). The effect of spousal violence on women's health: findings from the Stree Arogya Shodh in Goa, India. J Postgrad Med.

[12] Garcia-Moreno C (2002). International conference on ‘the role of health professionals in addressing violence against women’: an overview. International journal of gynaecology and obstetrics: the official organ of the International Federation of Gynaecology and Obstetrics.

[13] Miller E, Tancredi DJ, Decker MR, et al. A family planning clinic-based intervention to address reproductive coercion: a cluster randomized controlled trial. Contraception. 2016; 10.1016/j.contraception.2016.02.009. [published Online First: 2016/02/20] 10.1016/j.contraception.2016.02.009 PMC4884549 26892333

[14] Mahapatro M, Gupta RN, Gupta V, et al. Domestic violence during pregnancy in India. Journal of interpersonal violence. 2011;26(15):2973–90. 10.1177/0886260510390948. [published Online First: 2011/02/02] 10.1177/0886260510390948 21282118

[15] Christofides N, Jewkes R. Acceptability of universal screening for intimate partner violence in voluntary HIV testing and counseling services in South Africa and service implications. AIDS Care. 2010;22(3):279–85. 10.1080/09540120903193617. [published Online First: 2010/04/15] 10.1080/09540120903193617 20390507

[16] Undie CC, Maternowska MC, Mak'anyengo M, et al. Is routine screening for intimate partner violence feasible in public health care settings in Kenya? Journal of interpersonal violence. 2016;31(2):282–301. 10.1177/0886260514555724. [published Online First: 2014/11/09] 10.1177/0886260514555724 25381272

[17] John IA LS AO. Acceptance of screening for intimate partner violence, actual screening and satisfaction with care amongst female clients visiting a health facility in Kano, Nigeria. Afr J Prm Health Care Fam Med. 2011:3(1). 10.4102/phcfm.v3i1.174.

[18] Divya Nair AM, Ramakrishnan J, Mahalakshmy T, Sahu SK. Feasibility of routine screening for domestic violence among women attending an urban health center in Puducherry, India. International Journal of Medical Science and Public Health. 2015;4(9):1191–6. 10.5455/ijmsph.2015.15032015242.

[19] Miller E, Silverman JG. Reproductive coercion and partner violence: implications for clinical assessment of unintended pregnancy. Expert review of obstetrics & gynecology 2010;5(5):511-515. doi: 10.1586/eog.10.44 [published Online First: 2010/09/01]. 10.1586/eog.10.44 PMC3282154 22355296

[20] Taket A, Nurse J, Smith K, et al. Routinely asking women about domestic violence in health settings. BMJ Clinical research. 2003;327(7416):673–6. 10.1136/bmj.327.7416.673. [published Online First: 2003/09/23] 10.1136/bmj.327.7416.673 PMC196400 14500444

