# Supplementary material for: Gender-based violence screening methods preferred by women visiting a public hospital in Pune, India
Source: BMC Womens Health. 2018 Jan 15;18:19. doi: 10.1186/s12905-018-0515-2 (PMC5769341; doi:10.1186/s12905-018-0515-2)
Supplement: Additional file 1: — Gender Based Violence Questionnaire. (PDF 471 kb) [file 12905_2018_515_MOESM1_ESM.pdf]

Gender-Based violence

PID:

1. What is your age? ☐☐ Yrs.

2. Are you currently:

☐ 1. Deserted ☐ 2. Divorced ☐ 3. Married ☐ 4. Unmarried ☐ 5. Widowed

☐ 6. Refused to Answer

3. What is your mother tongue?

☐ 1. Marathi ☐ 2. Hindi ☐ 3. Other . ________________

4. What is your family type?

☐ 1. Nuclear ☐ 2. Joint

5. How many years of education have you had?

☐ 01.None ☐ 02.Primary (1-4) ☐ 03.Secondary (6-10) ☐ 04.Higher (11+12)

☐ 05.College (13-16) ☐ 06. 16 above ☐ 66. Refused to answer

6. What is your occupation:

☐ Housewife ☐ Unskilled manual worker ☐ Professional ☐ Skilled manual worker/farmer

☐ Other (b. Specify __________________)

Acceptability and knowledge of GBV support organizations

1. Are you aware of any organizations or individuals that exist to help women who have experienced abuse?

   a. ☐ Yes ☐ b. No

   If yes: Name of organization 1. ....

   2. ....

   3. ....

   (Probe for legal support, police reporting, social support, safety planning)

2. If yes, how comfortable are women in accessing those services?

   a. They hardly access those services ☐

   b. They access services only if they have threat to their lives. ☐

   c. They fear if they access the service they will face more violence ☐

   d. Other............................ ☐

Experiences with screening and acceptability of screening for GBV

3. How comfortable are women in talking about violence experiences with their health providers?

    ....
    ....
    ....
    ....

4. Think about your experiences getting health care, have any of your doctors or nurses asked you about violence experiences?

    a. ☐Yes b. ☐ No

    If yes, how comfortable did it feel for you?

    a. I did not feel comfortable at all. ☐
    b. I liked it as I felt someone is there to ask about it ☐
    c. I was scared, if my family (husband/in laws will come to know) ☐
    d. Other... ☐

5. Do you think women want to be asked about abuse experiences at health care facility?

    Yes ☐ No ☐

    for any answer ask reason..

6. Would women answer honestly about abuse experiences?

    Yes ☐ No ☐

    If No? why...

    Would they be more comfortable being asked face-to-face, or completing a survey or computerized tool?

    a. Face to face ☐
    b. Completing survey ☐
    c. computerized tool ☐

    ....

7. Who is the best person to ask women for violence? With whom will women be most comfortable or open?

    a. Doctor 1.Male ☐
    2. Female ☐

    b. nurse, ☐
    c. community health worker ☐
    d. Other................. ☐

    ....
    ....

....
....

8. Have you ever been hit, pushed, slapped, choked or otherwise physically hurt by your husband or someone you were in a sexual relationship with?

a. ☐ Yes ☐ b. No

8a. If yes, has this happened in the past 12 months?

a. ☐ Yes ☐ b. No

9. **H**ave you ever had sex (vaginal, oral, or anal sex) when you didn't want to because your husband/partner insisted or pressured you in a way you felt you could not refuse, but didn't use force or threats? (Choose one)

a. ☐ Yes ☐ b. No

2a. If yes, has this happened in the past 12 months?

a. ☐ Yes ☐ b. No

10. Have you ever had sex (vaginal, oral, or anal sex) with your husband/partner when you didn't want to because your partner used force or threats?

a. ☐ Yes b. ☐ No

3aIf yes, has this happened in the past 12 months?

a. ☐ Yes ☐ b. No

11. Did you face any humiliation by your husband/partner in front of others?

a. ☐ Yes b. ☐ No

If yes, how many times this happened in the past 12 months?...........

12. I think it is helpful for health care providers to talk about healthy and unhealthy relationships, including violence, with patients.

a. ☐ Strongly agree b. ☐ Agree c. ☐ Neutral d. ☐ Disagree
e. ☐ Strongly disagree

13. I would feel comfortable if my provider asked me about my relationships, including violence.

a. ☐ Strongly agree b. ☐ Agree c. ☐ Neutral d. ☐ Disagree
e. ☐ Strongly disagree

14. Comments of Interviewer:

....
....
....
....
....
....
